# Hepatitis Vaccines

**DOI:** 10.3390/vaccines4010006

**Published:** 2016-03-11

**Authors:** Sina Ogholikhan, Kathleen B. Schwarz

**Affiliations:** Division of Pediatric Gastroenterology and Nutrition, Pediatric Liver Center, Johns Hopkins Medical Institutions, CMSC 2-125, 600 North Wolfe Street, Baltimore, MD 21287, USA; sogholi1@jhmi.edu

**Keywords:** hepatitis A, B, C, D, and E, vaccines

## Abstract

Viral hepatitis is a serious health problem all over the world. However, the reduction of the morbidity and mortality due to vaccinations against hepatitis A and hepatitis B has been a major component in the overall reduction in vaccine preventable diseases. We will discuss the epidemiology, vaccine development, and post-vaccination effects of the hepatitis A and B virus. In addition, we discuss attempts to provide hepatitis D vaccine for the 350 million individuals infected with hepatitis B globally. Given the lack of a hepatitis C vaccine, the many challenges facing the production of a hepatitis C vaccine will be shown, along with current and former vaccination trials. As there is no current FDA-approved hepatitis E vaccine, we will present vaccination data that is available in the rest of the world. Finally, we will discuss the existing challenges and questions facing future endeavors for each of the hepatitis viruses, with efforts continuing to focus on dramatically reducing the morbidity and mortality associated with these serious infections of the liver.

## 1. Introduction 

According to the Centers for Disease Control and Prevention, the reduction in morbidity and mortality associated with vaccine-preventable diseases in the United States has been described as “one of the 10 greatest public health achievements of the first decade of the 21st century [1].” A recent analysis concluded that routine childhood vaccination will prevent 322 million cases of disease and about 732,000 early deaths among children born during 1994–2013, for a net societal cost savings of $1.38 trillion. [1] Since safe and effective vaccines for hepatitis A and B for children have been available for years, these vaccines undoubtedly contribute to the reductions in morbidity and mortality associated with vaccine-preventable diseases in the United States. In this section dedicated to hepatitis vaccines, we will focus on vaccine development, the impact of vaccines on disease burden, and challenges remaining for the hepatitis A and B vaccines. Given that there is no available vaccine for the hepatitis C virus, we will discuss the problematic pathway toward vaccine development for that virus. For hepatitis E there is no FDA-approved vaccine, so we will present preliminary data on existing vaccines from countries outside the United States. Given that viral hepatitis A, B, C, D, and E together constitute the eighth leading cause of mortality worldwide, resulting in an estimated 1.44 million deaths in 2010 (HIV is the sixth and tuberculosis the 11th) [2], it is clear that safe and effective vaccines for all of these viruses as well as enlightened and enforced public policy to insure widespread dissemination could dramatically reduce the morbidity and mortality associated with these serious infections of the liver. An in-depth discussion of the molecular virology, epidemiology, and history of viral discovery of each of the hepatitis viruses is beyond the scope of this paper. For these topics the reader is referred to the comprehensive textbook, *Viral Hepatitis*, 4th edition, edited by Thomas *et al*. [3].

## 2. Hepatitis A Virus (HAV)

**Figure 1:**
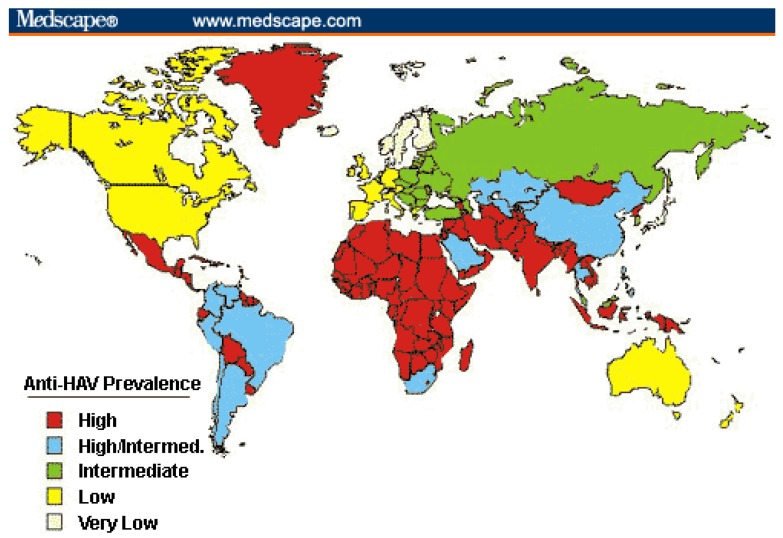
Global prevalence of the hepatitis A virus. Used by permission of the publisher.

In the pediatric population, HAV is one of the most common vaccine-preventable diseases. Although HAV is a self-limiting disease of the liver, it can be severe, leading to an estimated 34,000 deaths in 2005 worldwide [3]. HAV is transmitted primarily by person-to-person contact via the fecal-oral route along with contaminated food or water. How severe an acute Hepatitis A infection will be is strongly dependent on age. Acute infection in children below the age of five years is asymptomatic in 80%–95% of the cases. However, in adults, 70%–95% of infections result in some form of symptomatology. Roughly 1% of cases result in acute liver failure among children [4]. Half of these cases go on to require liver transplantation, while the other half make a full recovery. The incidence of HAV in unprotected travelers is about 3 per 1000 travelers according to the WHO data [5]. HAV is most prevalent in areas with poor hygiene or sanitation (see Figure 1). As a result, most children living in such places are infected before they reach adulthood. Due to most children being asymptomatic in these areas, HAV is often underreported [5].

### 2.1. History of Vaccine Development

Since the 1990s, several vaccines against HAV have been commercially available, including both an inactivated and live attenuated vaccine. The first inactivated HAV vaccine was produced from a strain of the virus propagated in cell culture. This was then purified and inactivated using formalin and the purified HAV strain was then grown on human diploid MRC-5 cells [6]. This vaccine was clear of any remaining infective capability *in vitro*. Marmoset monkeys injected with the vaccine did not show any changes in hematological or chemistry values, effectively demonstrating vaccine administration without the clinical effects of an acute HAV infection. Infectious HAV particles were detected neither in feces nor in the sera of the vaccinated primates by enzyme-linked immunosorbent assay. With regards to the immunogenicity of the vaccine, guinea pigs were injected with 0.8, 0.2, or 0.05 micrograms of HAV antigen. The antibody response was dose dependent, with one injection of 0.2 micrograms of the HAV antigen prompting sero-conversion in 100% of animals [7]. Further increase of antibody titers was achieved after the second and third immunizations. These initial tests showed this methodology was safe and provided a great immunologic response in the animal models, which led to further testing in human subjects confirming the same results [8].

Since the late 1970s, the development of the formalin-inactivated hepatitis A vaccine, Vaqta^®^, resulted in the commencement of Phase I clinical studies in 1989 and progressed to Phase III clinical trials [8]. These trials demonstrated efficacy of a single dose of the vaccine in preventing clinical HAV disease in pediatric populations in Monroe, NY. Inactivated HAV vaccines have proven to be among the most immunogenic, safe, and well-tolerated vaccines [8]. The persistence of antibody in adults more than 25 years after vaccination is estimated to be >95 percent [9]. The HAV vaccine has also proven to be an extremely safe vaccination. In a study of safety and efficacy, there were no vaccine-related, serious adverse events reported in approximately 40,000 children who participated [10].

There are currently four inactivated monovalent HAV vaccines available (Havrix^®^, Vaqta^®^, Avaxim^®^, and Epaxal^®^). Only Havrix and Vaqta are available in the United States. These all include antigens using differing strains of the virus. It is important to note that all four vaccines are of equal efficacy in humans. Combined vaccines that include hepatitis A and B or hepatitis A and typhoid have been developed and used for adults intending foreign travel [5].

It is important to note the use of immune globulin (Ig) against HAV infection. This is a well-studied topic, with dosing recommendations at documented 0.02 mL/kg body weight (post exposure prophylaxis) and 0.02 and 0.06 mL/kg body weight pre exposure prophylaxis for travel <3 months (single injection) and >5 months respectively (in which case it should be given every five months) [5]. Ig must be injected no later than 14 days post-exposure and has been shown to have 80%–90% efficacy. However, the use of immune globulin is on the decline worldwide mostly due to low concentrations of anti-HAV IgG along with the lower cost of vaccination, which can induce rapid protection after one dose [11].

### 2.2. Recommendations for Administration of HAV Vaccine

Initially, in 1996, the Advisory Committee on Immunization Practices (ACIP) of the American Academy of Pediatrics recommended routine immunization of selected groups at high risk of HAV such as those with chronic liver disease, human immunodeficiency virus (HIV) infection, or illicit drug use. Subsequently, in 1999, the ACIP expanded its recommendations to include immunization for children >2 years of age living in areas with high disease incidence. However, in 2006, the recommendations changed to immunization of all children at one year of age in the United States. Inactivated HAV vaccines are licensed for use in people ≥12 months of age. According to the manufacturers, a complete vaccination schedule consists of two doses administered into the deltoid muscle. The interval between the first (primary) and second (booster) dose is commonly 6–12 months; however, the interval can be extended to 18–36 months.

Globally, the World Health Organization (WHO) recommends immunization for children aged ≥1 year in places with intermediate endemicity (see Table 1). In locations with high endemicity, universal vaccination is not recommended as almost all of the inhabitants are asymptomatically infected with HAV during childhood. Infection with HAV confers life-long immunity [5]. Recommendations for adult vaccination are given in Table 1.

### 2.3. Impact of Vaccine on Disease Burden

The World Health Organization (WHO) estimates an annual total of 1.5 million clinical cases of HAV worldwide; however, this is believed to be greatly underreported as sero-prevalence data indicates that tens of millions of HAV infections occur each year [12]. In the United States in the 1980s–1990s, an average of 26,000 acute HAV cases were reported per year, representing approximately 270,000 infections annually [12]. Since licensure of effective HAV vaccine in the mid-1990s, U.S. HAV rates have fallen since 1999, when routine childhood vaccination was initiated. By 2004, the overall rate had declined to 1.9/100,000 population, the lowest rate ever recorded and 79% lower than previously recorded [13]. The effectiveness of inactivated HAV vaccines was shown in large-scale immunization programs in North American populations, resulting in 94%–97% reduction in the incidence of acute HAV within 6–10 years. These marked declines occurred with relatively modest vaccination coverage, as shown in vaccination survey of adolescents in 2009 that showed only a 42% national one-dose vaccination rate, suggesting that strong herd immunity accompanies the initiation of routine vaccination programs [14]. Routine childhood vaccination has produced similar results in Israel and selected regions of Italy, Spain, and Australia [15,16,17]. HAV vaccination is currently a low public health priority for many resource-limited countries where most persons are asymptomatic at the time of infection in childhood. However, shifts in the epidemiologic patterns of disease have been seen with improved global sanitation and clean water projects leading the way in improved hygiene. In the future, strategically targeted HAV vaccination of children could produce substantial public health benefits in these regions. It is important to note that increased vaccination among children in high prevalence areas would likely result in acute infections among an older population.

### 2.4. Remaining Challenges

Vaccination against HAV should be part of a comprehensive plan for the prevention and control of viral hepatitis. A first priority should be continued improvement of overall sanitation levels and hygiene. During outbreaks of HAV, measures to assess overall immunization status and sanitation conditions in those areas will be important. Countries should collect and review the information needed to estimate their national burden of HAV. Implementation of registration systems, acute disease surveillance, and capturing cases of fulminant hepatic failure and/or causes of liver transplantation should also continue to aid in targeting areas in need of expanding vaccination efforts. Economic evaluation, including cost-effectiveness analyses of relevant immunization strategies, can serve as a useful additional element for decision-making to supplement previous analyses [18]. Several large-scale cost-effectiveness analyses have demonstrated that there should also be continued efforts in assessing lifelong immunity with regard to single-dose and two-dose vaccination regimens. Although mathematical estimates predict immunity up to 25 years and the data do not support adulthood boosters, there has not been enough time since the start of routine childhood examinations to fully assess these estimates.

## 3. Hepatitis B Virus (HBV)

HBV is a major cause of morbidity and mortality worldwide. About two billion people have been infected by HBV at some time; 370 million are currently chronically infected and around one million die each year from HBV-related liver diseases, particularly liver cancer [19]. HBV contains eight different genotypes (A–H). The most effective way of reducing global incidence is vaccination supported by effective enforceable public health policies.

### 3.1. History of Vaccine Development

The first HBV vaccine to be developed was a subviral particle (hepatitis B surface antigen) purified from the inactivated plasma of asymptomatic carriers of HBV [20,21]. This was followed by a vaccine developed from cloned HBV DNA fragments encoding the S protein, first in mouse L cells and then in yeast cells [22,23]. Both plasma-derived and cell-based vaccines are safe and effective and have been utilized since the 1990s in some but not all parts of the world.

### 3.2. Recommendations for Administration of HBV Vaccine 

The schedules for the vaccine have been developed by the Advisory Committee on Immunization Practices (ACIP) and apply to infants, children, adolescents, and high-risk adults [24]. ACIP recommends universal testing of pregnant mothers for HBsAg. Vaccination dose and schedule is the same despite the maternal HBsAg status. If the maternal status is HBsAg positive, then hepatitis B immunoglobulin (HBIG) is also given. The schedules also differ as to whether the vaccine used is a monovalent vaccine or one of the combination vaccines (Pediarix^®^––diphtheria, pertussis, tetanus, polio, and HBV) and Comrix^®^ (*Haemophilus influenzae* and HBV). Twinrix^®^ (for HAV and HBV) is available for children 1–15 years of age in Europe. It is not approved by the FDA for use in children in the United States. However, the Twinrix vaccine is FDA approved for adults 18 years of age and older. The schedules also differ among countries and sub-populations. There are also separate recommendations for preterm infants and a caution that topical administration of EMLA cream should be done prior to administration of HBV vaccine in this population [25] to minimize the pain associated with the vaccine in these infants. It should also be noted that HBV vaccination is equally efficacious in non-infected infants, children, and adults. Please see Table 2 for adult vaccination recommendations. A commonly asked question is the duration of the HBV vaccine’s effect. Keck *et al.* [26] showed that the majority of vaccines are antibody positive 7–9 years after the initial vaccine course, although only about half had levels of HBsAg >10 IU/mL, which are thought to be protective. 

### 3.3. Impact of the Vaccine on Disease Burden

**Figure 2:**
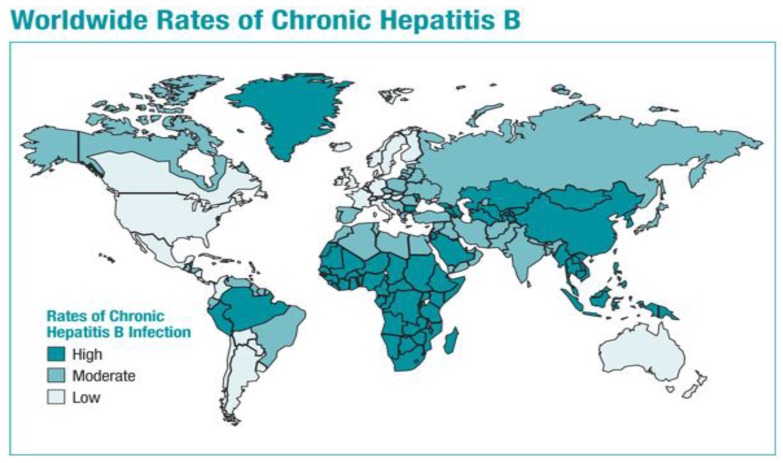
Global prevalence of HBV [24]. Figure is used with permission from the publisher.

Despite the development of safe and effective HBV vaccines over three decades ago, HBV remains a major public health problem worldwide, the leading cause of liver cancer, and an important contributor to cirrhosis as well as end-stage liver disease requiring liver transplantation. A recent systematic review estimated the HBsAg prevalence globally for the years 1990 and 2005 [27]. The total number of HBsAg positive (CHB) subjects in 2005 was estimated at 240 million, higher than the 223 million estimated for 1990. The global prevalence in 2005 was 3.7% *vs.* 4.2% for 1990. As shown in Figure 2, the areas of highest prevalence in the world are Asia and Africa. Locarnini *et al.* [28] have argued that effective and enforced public health policies are needed in order to actualize a universal HBV vaccine and to see the marked reduction in morbidity and mortality that would be possible if this vision were to become a reality. As of 2008, 177 of 193 WHO member states (92%) had integrated HBV vaccination into their national infant vaccination schedules [24]. 

**Figure 3:**
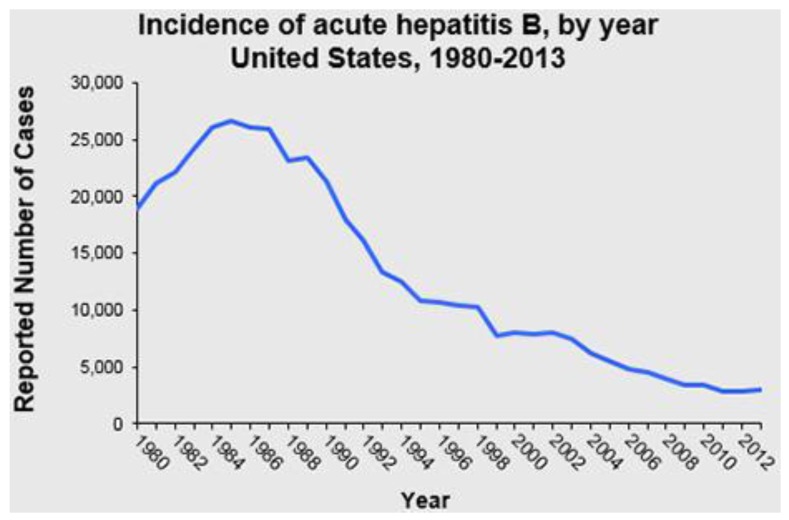
Reported acute hepatitis B incidence, United States, 1980-2013 [1]. Figure is used with permission from the publisher.

In contrast, in the United States the aggressive policy of universal vaccination of infants, children, adolescents, and high-risk adults initiated in the early 1990s was followed by a dramatic decline in acute HBV incidence (Figure 3), demonstrating that if the vaccine is made widely available it can have great public health benefits. Evaluating the introduction of childhood vaccination, various countries showed a marked decline in disease burden; however, other health programs were also instilled including behavioral changes in at-risk groups such as a needle exchange program [29]. In 2005 the ACIP published their updated recommendations, which undoubtedly hastened the decline of acute HBV infections in the United States [24]. These recommendations included universal infant vaccination beginning at birth, and increasing vaccine coverage among previously unvaccinated children and adolescents. Multiple strategies were used to implement these recommendations including (1) establishing standing orders for HBV vaccine beginning at birth; (2) delivery hospital policies for identification of and administration of immunoprophylaxis to infants born to mothers who are hepatitis B surface antigen (HBsAg) positive and to mothers with unknown HBsAg status at the time of delivery; and (3) vaccination record reviews for all children aged 11–12 years and children and adolescents aged <19 years who were born in countries with intermediate and high levels of HBV endemicity, HBV vaccine requirements for school entry, and focusing on ways to improve coverage of adolescents. In 2014, the CDC reported that 91.6% of infants and children in the United States had received three or more doses of HBV vaccine, although <90% had received the recommended birth dose of the vaccine. In general, coverage was worse in children living in poverty [1].

### 3.4. Remaining Challenges

The remaining challenges fall into three areas: issues special to infant vaccination, public health problems related to poor adherence to completion of vaccine schedules, and biological issues related to inadequate responses to existing vaccines and ways to increase immunogenicity.

While lauding efforts to improve HBV vaccine delivery to all newborns around the world, Thio and colleagues [30] have called attention to the very real problem that thousands of infants are born at home and are unlikely to receive the birth dose of vaccine even in those countries that endorse it. The authors are recommending that strong consideration be given to the additional strategy of prenatal treatment to reduce HBV viral load, a strategy that has already been shown to be effective for HBV-infected mothers [31]. In the special situation in which the father is HBsAg positive although the mother is not, it has been shown that vaccination of pregnant mothers does decrease HBV transmission from father to infant as long as the mother develops anti-HBsAb [32]. A small percentage of vaccinated infants do develop occult HBV, but this percentage is much lower for infants who are vaccinated *vs.* those who are not [33]. Two practical issues related to HBV infant vaccination are the poor follow-up of vaccinated infants born to HBsAg+ mothers [34] and the possibility of side effects from earlier vaccines such as speech delay in infants given vaccines containing thiomersal during 1991–2001 [35]. This issue, however, is no longer relevant as this ingredient is not part of the current vaccines.

Another practical issue in vaccine delivery is the poor adherence to vaccine schedules in patients with chronic conditions such as diabetes [36]. Other populations in which it has proved difficult to achieve high rates of vaccine coverage include groups at high risk for HBV such as the homeless or incarcerated [37] and, interestingly, medical students [38].

Populations that have been shown to have impaired vaccine responses include adults >55 years of age and injection drug users for whom an accelerated vaccine schedule looks promising [39,40]. For patients with HIV-1, strong consideration should be given to administering a double dose [41], although some have questioned the necessity of that approach. While patients with inflammatory bowel disease treated with immunosuppressants [42], on hemodialysis [43], or in renal failure [44] respond poorly to the HBV vaccine, kidney transplant recipients seem to respond well [45]. The current recommendation for patients who have undergone liver transplantation for HBV-related liver disease who have subsequently been vaccinated to prevent reinfection is to verify the absence of cccDNA in peripheral blood mononuclear cells and the liver prior to withdrawal and immunoprophylaxis with HBIG and/or antiviral agents [46]. Hepatitis D virus (HDV) vaccine attempts have been unsuccessful, unfortunately, since HIV and intravenous drug users (IDU) constitute a reservoir of HDV infection [47]. Unfortunately, the current anti-HDV vaccines have not elicited a strong enough T cell response in infected woodchucks to prevent the spread of HIV, so the challenge is to use this animal model to develop more potent vaccines that can then be tested in humans.

The search is ongoing for more potent HBV vaccines. A hyaluronic acid-based combination is promising [48], as are a variety of other old and new adjuvants including TLR9 [49]. Investigators are working on nasal HBV vaccines as a strategy to enhance mucosal immunity and decrease sexual transmission of the virus [50]. GMCSF has been explored both as a vaccine adjuvant and for developing a therapeutic vaccine [51]. Other therapeutic vaccines tested thus far have either been HBV protein vaccines (largely unsuccessful) or DNA vaccines targeting hepadnaviral proteins to induce immune responses and enhance viral clearance, especially to break immune tolerance in chronically infected animals. The most effective approach thus far in preclinical studies of therapeutic DNA vaccines appears to be the delivery of HBV DNA by *in vivo* electroporation. Various other strategies include genetic adjuvants, use in tandem with antiviral drugs to lower the viral load, prime-boost regimens, and delivery via plasmids. Trials in various animal models (mouse, chimpanzee, duck, and woodchuck) have been promising and clinical trials in humans are underway [52]. In some chronically infected woodchucks, Liu *et al.* demonstrated that it was possible to achieve complete viral clearance with a combination of therapeutic DNA vaccination, the nucleoside analogue entecavir, and inhibiting of the inhibitory receptor programmed cell death-1 (PD-1), which enhanced virus-specific T cell immunity [53]. A final challenge to the development of universally potent and effective HBV vaccine is the presence of vaccine escape mutants. Neutralizing (protective) antibodies (anti-HBs) induced by vaccination are targeted largely towards the amino acid hydrophilic region of the HBsAg. This region is referred to as the common “a” determinant spanning amino acids 124–149. Antibodies directed to the determinant provide protection against all HBV genotypes (from A to H). This neutralizing antibody is responsible for the broad immunity afforded by hepatitis B vaccination. A group of Italian children was found to have a mutation in a determinant and thus developed infection despite vaccination [54]. Another problem affecting effective universal HBV vaccine is the development of pol mutants during antiviral therapy. These pol mutants could in theory affect the part of the S gene that overlaps with the pol gene and thus be another source of HBV vaccine escape mutants. The reason for this is that the pol gene that encodes the polymerase completely overlaps with that part of the s gene that encodes the surface antigen; thus changes in each gene may affect the other. There are at least a dozen pol mutants associated with s mutants. HBV s mutants can infect individuals successfully vaccinated with the traditional recombinant surface antigen vaccine [55].

In summary, the achievements of the last 30 years in developing generally safe and effective HBV vaccines are to be enthusiastically lauded. The challenges for the future involve a multi-pronged approach combining biology, immunology, psychology, and public health policy. However, the knowledge available in 2015 provides a powerful infrastructure to make major progress in reducing the major global disease burden secondary to HBV. It is important to note that the prophylactic HBV vaccine is the first vaccine against a major human cancer.

## 4. Hepatitis C Virus

**Figure 4:**
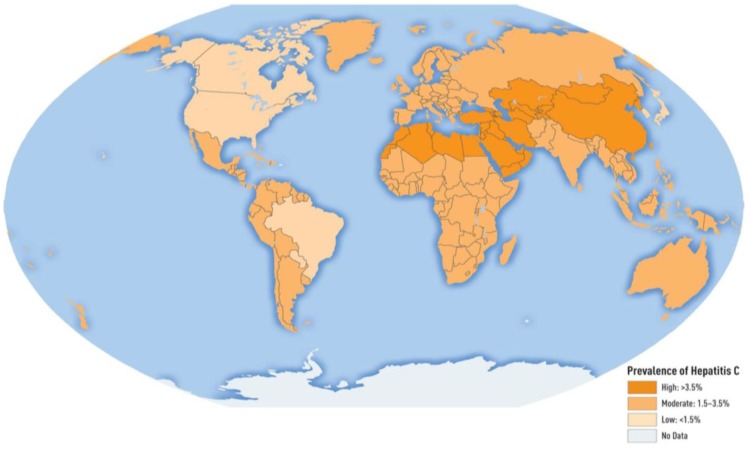
Global prevalence of hepatitis C virus [65]. This image was used with permission of the publisher.

The hepatitis C virus (HCV) is a major cause of morbidity and mortality worldwide and affects approximately 3% of the world’s population. It is a blood-borne virus with a global distribution of 185 million people [56]. The global prevalence is shown in Figure 4. Available estimates indicate that worldwide there were 54,000 deaths and 955,000 disability adjusted life-years associated with acute HCV infection. However, the major burden from HCV infection comes from the sequelae associated with chronic infection [57]. Nearly three to four million people are newly infected each year per estimates; the remainder are chronically infected and at risk of developing liver diseases including cirrhosis and liver cancer. An estimated 350,000 deaths occur each year due to HCV-related sequelae [57]. HCV is the leading cause of liver cancer and end-stage liver disease, requiring liver transplantation in many countries. HCV has seven genotypes in all with subtypes among the various genotypes. Over the entire HCV genome, individual genotypes differ at between 30%–35% of nucleotide sites, with 20%–25% nucleotide variability apparent between subtypes [58]. Understanding of and research on the various genotypes is vital to vaccine development. While vaccine development has been progressing over the years, interferon therapies have been the cornerstone of HCV treatment for decades; however, there have been many side effects associated with interferon therapy. HCV therapy has greatly improved in the last several years with the development of new directly acting antivirals (DAAs), which are targeted at specific steps within the HCV life cycle. DAAs have success rates >95%, dramatically superior to interferon therapy. However, a significant cost burden and an inability to prevent re-infection are disadvantages to DAA therapy. An additional challenge is the remaining concern for continued liver damage, even after a cure [59]. Although most liver damage does stabilize and slowly correct, there is concern about a point of no return, requiring continued monitoring of hepatic laboratory tests and medical management. Chronic hepatitis C infection can be cured, resulting in a virus-free status. However, a potential therapeutic vaccination would need to be genotype specific in order to cure.

### 4.1. History of Vaccine Development

An effective vaccine against HCV would aim to replicate or accelerate the immune pathway during natural infection to prevent disease chronicity. With prior evidence of other vaccines almost completely eradicating serious endemic diseases such as smallpox and polio, vaccine development for HCV remains a hopeful option. However, vaccine development has been especially challenging for HCV due to several reasons. The most difficult reason is the vast diversity among the genetic sequence of the seven different known genotypes of HCV. Other barriers include the lack of appropriate animal models since the use of non-human primate models is challenging to obtain. The numerous mechanisms used by the HCV to avoid detection by the innate immune system have also been highly challenging in addition to its extreme genetic diversity. Vaccine development and prevention of disease would be most beneficial for the at-risk population, *i.e.*, uninfected IV drug users and nosocomial infections without the need for DAAs or other costly therapies. There is already evidence of spontaneous clearance of primary HCV among patients in up to 25% of cases [60,61]. Generated HCV antibodies are generally ineffective at clearing viral infection.

Extensive T cell involvement is needed for spontaneous HCV clearance in acutely infected persons, demonstrating that a vaccine would need to generate both CD4+ and CD8+ responses [62]. However, reinfection of HCV shows a different immune response when compared to the primary infection, with a broader and more rapid cellular immune response along with cross-reactive antibodies [62]. There have been several vaccine candidates that have been tested in primates and only a small subset in the human population. In a proof of concept study of a therapeutic vaccine, there was demonstration of clear interest in the ChronVac-C prime/MVATG16643 boost strategy, in agreement with reports arguing in favor of heterologous vaccine approaches. This strategy used both the MVATG16643 vaccine (three injections one week apart) and the ChronVac-C vaccine (two injections four weeks apart), in which additive effects were seen [63].

However, only one study has had promising results in an at-risk population. This approach uses a replicative defective simian adenoviral vector (ChAd3) and a modified vaccinia Ankara (MVA) vector that encodes the NS3, NS4, NS5A, and NS5B proteins of HCV genotype 1b. HCV-specific T cells induced by ChAd3 are optimally enhanced with MVA, which generated very high levels of both CD8(+) and CD4 (+) HCV-specific T cells targeting multiple HCV antigens. Thus, a sustained memory and effector T cell populations were generated in this novel human study [64]. This vaccine is currently being evaluated in a phase I/II study with results expected in 2016 (see ClinicalTrials.gov NCT01436357). This study is a double-blind, randomized, placebo-controlled study of HCV-uninfected male and females who are active IDU aged 18 to 45 years. In Stage I, 68 evaluable subjects will be enrolled and then an interim analysis of safety data will be performed. A planned interim analysis of safety and immunogenicity will be conducted based on data through one week after receipt of the second vaccination. If no safety signal is detected and there is evidence of a measurable immune response to HCV then an additional 382 volunteers will be enrolled in stage 2 (see ClinicalTrials.gov NCT01436357). Please see Table 3 for additional HCV vaccination trials.

There have been several vaccination trials looking at both preventative and therapeutic vaccinations. Data provided on Clinicaltrials.gov are listed in the table below.

### 4.2. Remaining Questions

Although there have been exciting breakthroughs in antiviral agents showing great efficacy in HCV treatment, to fully control the global distribution of HCV infection a combination of effective antiviral treatment, better screening techniques, and advancement in preventative vaccinations measures will be necessary. However, if an effective vaccine is developed, who should be given the vaccine? Should it only be the at-risk population, pregnant women, or those with other risk factors for liver disease? Should a hepatitis C vaccine be incorporated into the childhood vaccination schedule? Once a viable vaccination is available, these questions will need to be systematically addressed with both cost-effectiveness and epidemiologic evaluations. Many of these questions can be addressed using mathematical models.

## 5. Hepatitis E Virus

**Figure 5:**
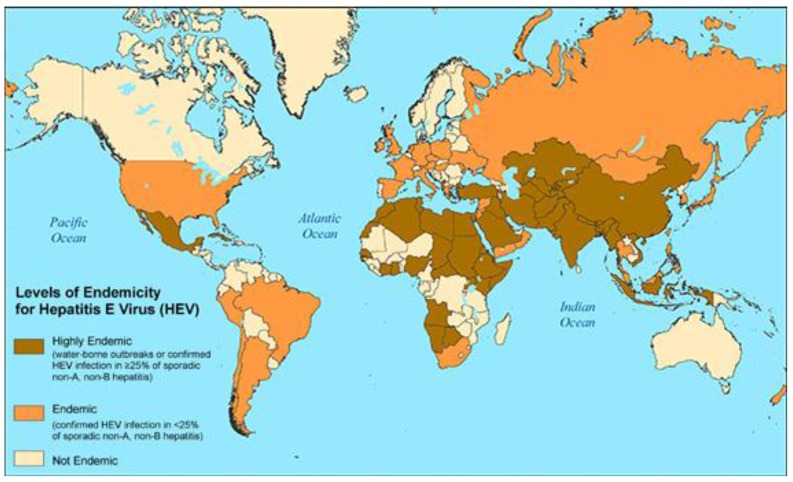
Global prevalence of HEV [70]

HEV was first discovered in 1983 [66]. For many years HEV was thought to be restricted to developing countries, was known to be an RNA virus causing short-lived disease, and was notorious for causing up to 30% mortality in pregnant women. Now emerging knowledge is that there are four genotypes and the first two are known to infect humans. Genotypes 3 and 4 were initially thought to be restricted to animal vectors. However, it is now apparent that immunosuppressed patients such as organ transplant recipients are not only susceptible to disease but the liver disease can be chronic and sometimes even fatal from all four genotypes [67]. Ribavirin treatment has been efficacious in some [67]. Patients with chronic liver disease may have high rates of morbidity and mortality [68]. The global disease burden is now recognized to be up to ~20 million with 70,000 fatalities/year [69]. Regions with prevalence rates >25% include Central America, large parts of Africa, and all of Asia (see Figure 5). Similar to HAV, improved water sanitation, personal and hand hygiene will be essential in decreasing these prevalence rates.

### 5.1. History of Vaccine Development

**Figure 6:**
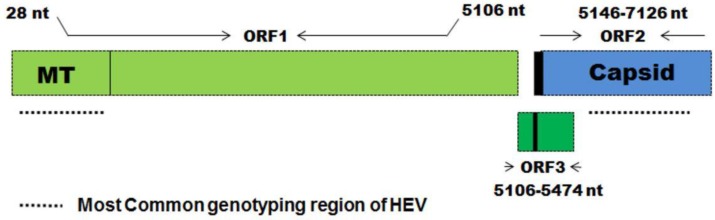
Hepatitis E viral genome [76]. This image was used with permission from the publisher.

Knowledge of the structure of the HEV genome has been essential for vaccine development [71]. The Open Reading Frame (ORF) 2 encodes the capsid protein against which all neutralizing antibodies are targeted (Figure 6). Therefore all of the efforts to develop a safe and effective vaccine have focused on ORF2 [72]. Vaccine antigens have been cloned in insects, yeast, baculovirus, *etc.* [73]. There have been a number of ORFs synthesized but to date only two have made it into clinical trials (see Table 4).

Haffer *et al.* [69] have comprehensively reviewed the two clinical trials of an HEV vaccine, the pertinent details of which are summarized in the Table 4. The first trial to be performed was a phase II trial in Nepal with prevention of clinical overt HEV disease as the endpoint. The vaccine was safe and highly effective. Unfortunately, it was not commercialized because it was not thought to be profitable. The larger trial was in China and again with a similar endpoint [74]. It was commercialized as Hecolin and is now in use, but only in China. Zhang *et al.* [75] recently published the follow-up study on the original cohort, demonstrating long-lasting immunity.

There has been much discussion of the need for a widely available safe and effective HEV vaccine for both routine use in national health programs and in outbreaks [78,79]. Recently the WHO organized a Special Advisory Group (SAGE) meeting in Geneva to review the status of the vaccine and make a recommendation about pre-qualification (*i.e.*, licensure for purchase by groups such as GAVI and UNICEF to use in humanitarian emergencies followed by epidemics of HEV infection, which occur regularly in South Asia and Sub-Saharan Africa). The WHO SAGE committee advised that further immunogenicity and safety studies were needed before the vaccine could be licensed. These included safety studies in pregnant women, children, patients with underlying chronic liver disease, and immune-suppressed patients. The SAGE committee also recommended the evaluation of the immunogenicity and efficacy of a more accelerated vaccine schedule that could be used in these high-risk populations or on the general population to prevent HEV during a humanitarian emergency. The report of the SAGE recommendations to WHO has been published in the *Bulletin of the WHO*.

### 5.2. Remaining Questions

Perhaps the major question is whether or not the vaccine should be used outside China and if it is not possible to gain access to the Chinese vaccine, what are the prospects for alternative products? [78] In addition, if a safe and effective HEV vaccine were to become available, who should receive it? [79] How long will the neutralizing protective antibodies last? [80] Is it safe and effective in young children? Pregnant women? [81] Immunosuppressed individuals? Those with chronic liver disease? [82] Should animals be vaccinated to prevent zoonotic transmission? [83] We predict that the next decade will bring answers to these questions and hope that these answers result in safe and effective vaccines that can have as dramatic an effect on disease burden as HAV vaccines have had globally.

## 6. Conclusions

In the last few decades, vaccines have contributed greatly to the goal of achieving control of viral hepatitis. A safe and effective vaccine against hepatitis A has translated into low rates of infection with this virus in North America, Europe, Australia, and New Zealand. However, the infection is endemic in most of the rest of the world. Challenges for the future include gathering more data to confirm the long-lasting immunity to hepatitis A virus induced by the vaccine as well as to develop better strategies to manage acute liver failure for hepatitis A. Introduction of a universal hepatitis B vaccine in the United States in the 1990s contributed to a marked decline in new cases of HBV. However, the number of people who are infected around the globe has continued to increase, likely due to an increasing global population. As a result, better public health strategies are urgently needed. Another area for future research related to the HBV vaccine is to develop more immunogenic vaccines for those who respond poorly to the current vaccines. It is also imperative to develop new therapeutic strategies for the millions of chronically infected individuals, such as therapeutic vaccines. A hepatitis D vaccine is also essential for the 350 million individuals infected with hepatitis B to prevent superinfection. There is no vaccine available at the present time to protect against infection with the hepatitis C virus; however, trials are currently underway. While there is a safe and effective anti-hepatitis E vaccine, at the present time it is only licensed for use in China. Given the recognition of HEV as a global pathogen, there is a need to develop HEV vaccines for use globally. Vaccines remain an important cornerstone in the battle to achieve dominance over hepatitis A, B, C, D, and E and thereby decrease morbidity and mortality from these infections in a cost-effective manner. 

## Figures and Tables

**Table 1:** Recommendations for adult vaccination [5].

Individuals traveling to or working in countries with high or intermediate rates of hepatitis A
Individuals with chronic liver disease
Individuals with clotting factor disorders
Men who have sex with men
Illicit drug users (injection and non-injection)
Individuals with close personal contact with an international adoptee from a country of high or intermediate endemicity during the first 60 days following arrival in the United States
Individuals working with HAV-infected primates or with HAV in a research laboratory
Individuals with recent exposure for post-exposure prophylaxis

**Table 2:** Recommendations for adult vaccination with hepatitis B vaccine.

**People at Risk of Infection by Sexual Exposure**
Sex partners of HBsAg-positive people
People with more than one sex partner in the past six months
People seeking evaluation or treatment for a sexually transmitted infection
Men who have sex with men
**People at Risk of Infection by Percutaneous or Mucosal Exposure to Blood**
Current or recent injection drug users
Household contacts of HBsAg-positive people
Residents and staff of facilities for people with developmental disabilities
Health-care workers with risk of exposure to blood-contaminated body fluids
People with end-stage renal disease
People with diabetes mellitus 19 through 59 years of age
**Others**
International travelers to regions with increased HBV prevalence
People with chronic liver disease
People with human immunodeficiency (HIV) infection

Modified from The Centers for Disease Control and Prevention [24].

**Table 3:** Clinical trials in HCV vaccine. All data below obtained from www.clinicaltrials.gov.

Study Type	Vaccine biology	Study Number	Individual type	Outcome
Preventative	AdCh3NSmut1 MVA-NSmut	NCT01436357	Healthy at risk population	Pending/Jan 2016
Preventative	AdCh3NSmut1 MVA-NSmut	NCT02362217	Healthy subjects/HIV +	Pending/Oct 2016
Therapeutic/peptide antigens	PEV2A PEV2B	NCT00445419	Hep C+	Updated 2/2010 – no results
Therapeutic	MRKAd5 HIV-1 gag vaccine	NCT00857311	Hep C+	Canceled - non-efficacious results of previous study using similar agent
Therapeutic	Recombinant IMPs Ad6NSmut and MVA-NSmu	NCT01701336	Chronic Hep C+	Last updated 2013, no results
Therapeutic	TG4040	NCT00449124	Chronic Hep C+	Withdrawn
Therapeutic	GI-5005	NCT00124215	Chronic Hep C+	Completed 2010 – no results
Therapeutic	IC41	NCT00601770	Chronic Hep C+	Phase 2 – no results 2014
Therapeutic – Genotype 1a	Autologous dendritic cells transduced with Ad encoding NS3	NCT02309086	Chronic Hep C+	Study complete 2015 – no results reported

**Table 4:** Clinical trials of HEV vaccine [69]. This figure was used with permission from the publisher.

	Shrestha *et al.* *NEJM*–March 2007 [74]	Zhu *et al.* *Lancet*–August 2010 [77]
Phase study	Phase II trial	Phase III trial
Type of study	Randomized, double-blind, placebo controlled	Randomized, double-blind, placebo controlled
Company	GlaxoSmithKline Biologicals	Xiamen Innovax Biotech
Country	Nepal	Jiangsu province, China
Recombinant protein ORF2	Baculovirus expressed 56 kDa	E. coli expressed HEV 239
Number of vaccines	1794 healthy subjects	112 604 healthy subjects
Randomization	HEV vaccine *vs.* placebo	HEV *vs.* HBV vaccine
Population	Mostly males (99.6%) Young (mean age 25 years)	Males and females, 16–65 years
HEV genotypes	Genotype 1 prevalent	Genotypes 1 and 4 prevalent with predominance
Doses	20 µg	30 µg
Route of administration	Intramuscularly	Intramuscularly
Intervals between doses	0, 1, 6 months	0, 1, 6 months
Primary end-point	Prevention of clinically overt HEV disease	Prevention of clinically overt HEV disease
Follow-up period	2 years post-vaccination	13 month post-vaccination
Efficacy		
After 1st dose	87.5%	95.5%
After 2nd dose	85.7%	100%
After 3rd dose	95.5%	100%
Side effects Commercialization	Increased injection-site pain Not further developed	No side effects Hecolin^®^ (Innovax)
